# Strain-resolved microbial community proteomics reveals simultaneous aerobic and anaerobic function during gastrointestinal tract colonization of a preterm infant

**DOI:** 10.3389/fmicb.2015.00654

**Published:** 2015-07-01

**Authors:** Brandon Brooks, Ryan S. Mueller, Jacque C. Young, Michael J. Morowitz, Robert L. Hettich, Jillian F. Banfield

**Affiliations:** ^1^Department of Earth and Planetary Sciences, University of California, BerkeleyBerkeley, CA, USA; ^2^Department of Microbiology, Oregon State UniversityCorvallis, OR, USA; ^3^Department of Genome Sciences and Technology, The University of Tennessee, KnoxvilleKnoxville, TN, USA; ^4^Chemical Sciences Division, Oak Ridge National LaboratoryOak Ridge, TN, USA; ^5^Department of Surgery, University of Pittsburgh School of MedicinePittsburgh, PA, USA

**Keywords:** colonization, infant gut, metaproteomics, microbiome, microbial ecology, physiology

## Abstract

While there has been growing interest in the gut microbiome in recent years, it remains unclear whether closely related species and strains have similar or distinct functional roles and if organisms capable of both aerobic and anaerobic growth do so simultaneously. To investigate these questions, we implemented a high-throughput mass spectrometry-based proteomics approach to identify proteins in fecal samples collected on days of life 13–21 from an infant born at 28 weeks gestation. No prior studies have coupled strain-resolved community metagenomics to proteomics for such a purpose. Sequences were manually curated to resolve the genomes of two strains of *Citrobacter* that were present during the later stage of colonization. Proteome extracts from fecal samples were processed via a nano-2D-LC-MS/MS and peptides were identified based on information predicted from the genome sequences for the dominant organisms, *Serratia* and the two *Citrobacter* strains. These organisms are facultative anaerobes, and proteomic information indicates the utilization of both aerobic and anaerobic metabolisms throughout the time series. This may indicate growth in distinct niches within the gastrointestinal tract. We uncovered differences in the physiology of coexisting *Citrobacter* strains, including differences in motility and chemotaxis functions. Additionally, for both *Citrobacter* strains we resolved a community-essential role in vitamin metabolism and a predominant role in propionate production. Finally, in this case study we detected differences between genome abundance and activity levels for the dominant populations. This underlines the value in layering proteomic information over genetic potential.

## Introduction

The human gastrointestinal tract (GIT) harbors a complex ecosystem of microorganisms, the microbiome, whose cell count outnumbers the cells of the human body by nearly ten to one (Smith, 2014). The genes of the microbiome encode byproducts critical for host health and development (Groer et al., 2014). Recent excitement in the field has been generated from findings implicating the microbial community in a variety of dysbioses from gut associated diseases like obesity and malnutrition (Turnbaugh et al., 2006; Smith et al., 2013), inflammatory bowel disease (Hold, 2014), and celiac disease (Nistal et al., 2012) to neurological disorders like depression (Park et al., 2013), anxiety (Diaz Heijtz et al., 2011), and autism (Hsiao et al., 2013). While significant contributions have been made to understand developed microbial communities in healthy and diseased adults, large gaps remain in understanding the acquisition of the human microbiome at birth, especially among preterm infants (Groer et al., 2014).

*In utero*, infants have a sparse microbiome (Ardissone et al., 2014), with the first major microbial inoculum encountered during the birthing process. Delivery mode, i.e., vaginal vs. cesarean section, can play a significant role in how a baby is colonized (Dominguez-Bello et al., 2010) as can dietary input, breast milk vs. formula (Guaraldi and Salvatori, 2012), and exposure to antibiotics (Groer et al., 2014). For example, infants born vaginally acquire a community more similar to the mother's vaginal and fecal microbiota, whereas infants born by cesarean section have a microbiome that is more similar to those of skin and hospital environments (Dominguez-Bello et al., 2010; Brooks et al., 2014). Cesarean section infants appear to have lower microbial richness and diversity relative to vaginally born infants at 4 months of age (Song et al., 2013). Throughout the first year of life the microbial community increases in diversity, reaching an adult-like state around 2.5 years of life (Koenig et al., 2011). The long term health effects of different colonization paths remains to be determined, but with the many direct and indirect effects of the microbiome, it is likely to play a critical role in the development of many diseases. Understanding dynamics that govern colonization, and ultimately defining a healthy colonization trajectory is critical, especially for preterm infants that are susceptible to numerous infections and developmental issues.

Very low birth weight (VLBW) infants accounted for approximately 35% of all infant deaths in 2009 (Groer et al., 2014). These infants have an increased risk for cardiorespiratory, hematological, intestinal, infectious, and neurological disorders (Groer et al., 2014). Most spend several months of their early lives in the neonatal intensive care unit (NICU), where administration of antibiotics is commonplace. Among VLBW infants, incidence rates of sepsis and necrotizing entercolitis (NEC) remain high (Bizzarro et al., 2014). Essentially all VLBW infants are characterized by low gut bacterial diversity and communities exhibit abrupt shifts in composition (which can be phage mediated), and an abundance of opportunistic pathogens (Sharon et al., 2013), relative to their full term counterparts. Many opportunistic pathogens in VLBW infants are facultative anaerobes. Typically during the first weeks of life, there is a shift from facultative to obligate anaerobes (Penders et al., 2006). Because facultative anaerobes are capable of growth with and without oxygen, their mode of growth cannot be determined from genomic sequence information alone. Further, organisms may be abundant but characterized by low activity levels, or vice versa. Here, we coupled strain-resolved community metagenomics data with mass spectrometry-based proteomics to resolve growth mode and to compare activity levels during colonization of a preterm infant. The samples collected during the first month of life for this VLBW infant were ideal for metaproteomic study because they had been genomically analyzed previously (Morowitz et al., 2010). Further, communities contained a limited number of highly abundant organisms, enabling deep proteomic analysis. We identified differences in metabolic potential and protein abundance levels in closely related strains, determined that both aerobic and a variety of anaerobic pathways were operational, and confirmed differences between genome abundance and metabolic activity.

## Materials and methods

### Infant description and sample collection

The female infant was delivered by cesarean section at 28 weeks gestation after premature rupture of membranes. The infant received antibiotics (ampicillin/gentamicin) for the first seven days of life (DOL). Breast milk enteral feeding was administered on DOL 4–9 but was stopped on DOL 9–13 because of abdominal distension. Enteral feeding was slowly resumed on DOL 13 with artificial formula (Similac Special Care 20/cal/fl oz; Abbott Nutrition). Additionally, parenteral nutrition was provided until caloric intake from enteral nutrition was adequate (DOL 28). Fresh fecal samples were collected on DOL 5–21 as available using a previously described technique (Morowitz et al., 2010). Samples used in this study were aliquots from the same fecal samples published previously (Morowitz et al., 2010). We refer to these samples here as phase three samples, since three distinct colonization phases were discernable from previously published community composition data (Morowitz et al., 2010). Informed parental consent was obtained before patient enrollment and research protocol approved by the Institutional Review Board of The University of Chicago (protocol # 15895A).

### Protein extraction, digestion, and nano-2D-LC-MS/MS

Protein extraction was conducted by boiling approximately 250 μg of fecal sample in 100 mM Tris-Cl containing 4% w/v SDS and 10 mM DTT, followed by bead beating for 30 min on a high setting. The supernatant was collected, boiled again, spun down, and precipitated overnight using 20% trichloroacetic acid at 80°C. Protein pellets were washed in acetone, re-solubilized with 8 M urea in 100 mM Tris-HCl pH 8, and sonicated for 5 min at 20% (10 s on, 10 s off) to break up the pellet. Iodoacetamide was added to block disulfide bond reformation. Between 1 and 3 mg of protein was then diluted to 4 M urea in 100 mM Tris-HCl pH8, and enzymatically digested using sequencing grade trypsin (Promega) for 4 h at room temperature. Peptides were diluted to 2 M urea, trypsin added, and digestion continued overnight. An acidic salt solution was used to wash the peptides (200 mM NaCl, 0.1% formic acid), which were then spun through a 10 kDa cutoff spin column filter (VWR).

In preparation for nanospray-two dimensional liquid chromatography coupled with tandem mass spectrometry (nano-2D-LC-MS/MS), a 150 μg peptide mixture was loaded onto a split-phase fused silica column containing reverse phase (C18) and strong cation exchange (SCX) materials. Samples were washed with solvent A (95% HPLC grade water, 5% acetonitrile, 0.1% formic acid) then solvent B (70% acetonitrile, 30% HPLC grade water, 0.1% formic acid). Peptides were placed in line with a nanospray emitter (New Objective) packed with reverse phase material then separated on-line using high performance two-dimensional liquid chromatography (McDonald et al., 2002; Washburn et al., 2002). Peptides were eluted by increasing ammonium acetate salt pulses as previously described (Ram et al., 2005; Lo et al., 2007; Verberkmoes et al., 2009), ionized via nanospray (200 nl/min) (Proxeon, Cambridge MA), and analyzed using an LTQ Orbitrap Velos mass spectrometer (Thermo Fisher Scientific, San Jose, CA). The mass spectrometer was run in data-dependent mode with the top 10 most abundant peptides in full MS selected for MS/MS, and dynamic exclusion enabled (repeat count = 1, 60 s exclusion duration). Full MS scans were collected in the Orbitrap at 30 K resolution. Two microscans were collected in centroid mode for both full and MS/MS scans. Technical duplicates (the same proteomic extract run on two separate nano-2D-LC-MS/MS runs) were run for all samples.

### Database composition, peptide matching, and read mapping

A search database was generated from manually curated genomes assembled from metagenomic reads previously published (Morowitz et al., 2010). Four taxa dominate these samples: *Serratia* (*UC1SER*), major and minor strain *Citrobacter* (*UC1CIT* and *UC1CITii*), and *Enterococcus* (*UC1ENC*). These taxa represent approximately 75% of the community composition, with the remaining 25% apportioned to several low ranking taxa (Morowitz et al., 2010). Low ranking taxa were excluded from the database to focus on organisms with higher peptide coverage. Strain-level variation between *Citrobacter* strains was resolved manually using Strainer version r-34 (Eppley et al., 2007). MS/MS spectra were searched against the concatenated database using MyriMatch version 2.1.111. The protein database is publicly accessible at http://ggkbase.berkeley.edu/UC1/, and the MS raw files have been uploaded to ProteomeXchange Consortium (http://proteomecentral.proteomexchange.org/) with the dataset identifier number PXD000114. Using metagenomic reads from data from previously published (Morowitz et al., 2010), read mapping to the updated strain-resolved metagenomic database was conducted using Bowtie 2 with sensitive parameters (Langmead and Salzberg, 2012).

### Pathway analysis

To summarize metabolic potential, we compiled lists of genes using annotation search terms implemented via the ggKbase lists function. List search terms were manually compiled, and made use of Enzyme Commission numbers (Bairoch, 2000), KEGG orthology numbers (Kanehisa et al., 2014), and other search terms to describe pathways. ggKbase is an online tool for genome binning, metabolic pathway curation and community composition analysis. The current dataset is available at http://ggkbase.berkeley.edu/UC1/. To easily visualize both metabolic potential and protein expression, an expression vs. potential ratio was plotted across all available lists and a subset of curated ggKbase lists (Supplemental Figure 1 and **Figure 3**, respectively). This ratio is the non-redundant count of features per list that were identified via proteomics, divided by the count of features per list. ggKbase lists are dynamic, so a static version linking genes to lists is available in Supplemental File 1.

## Results and discussion

### General proteome description

We characterized fecal proteome extracts from seven fecal samples via nano-2D-LC-MS/MS (DOL 13–21) and uniquely identified 1149–2636 microbial proteins per sample based on 4300–15370 distinct spectra. In total across all samples, we detected approximately 1000 proteins per organism for each of the three most abundant organisms. This represents coverage of 22% of the predicted proteomes of these organisms (Table 1). On average, approximately 550 proteins per organism were identified with unique peptides in each sample. DOL 13 samples exhibited extremely low peptide detection with unique spectral matches totaling 190 and proteins with unique spectral matches totaling 79. Thus, this sample was excluded from most analyses unless explicitly stated.

**Table 1:** **Genome and proteomics summary**.

**Taxa**	**UC1CIT**	**UC1CITii**	**UC1CITp**	**UC1ENC**	**UC1ENCp**	**UC1ENCv**	**UC1SER**
bp	4,902,348	4,901,982	59,966	2,576,397	77,038	37,230	5,027,440
Contigs	10	10	2	785	2	2	9
Max. contig	2,550,874	2,550,962	57,067	18,409	68,691	28,900	2,360,977
Genes	4829	4696	61	3589	94	56	4569
%GC	52	52	53	37	34	32	60
SCG (51 total)	50	48	0	40	0	0	45
Unique proteins detected	1049	1017	5	195	4	1	1021
Avg. unique protein per day	619	603	2	84	2	1	520
Unique protein matches	20,038	19,207	26	1636	25	17	16,129
Avg. unique protein matches per day	3331	3192	5	270	7	5	2677

### Microbial community profile and general functional characterization

**Figure 1:**
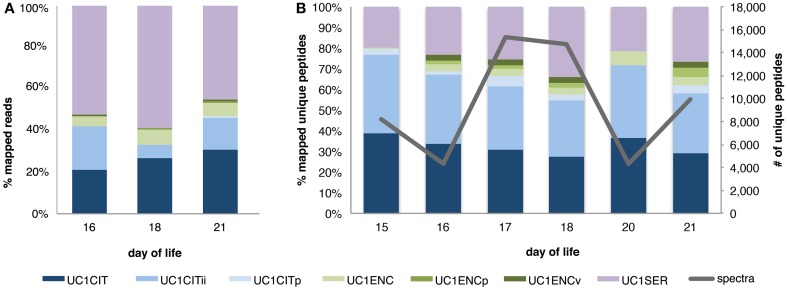
**Microbial community composition observed via read and peptide mapping**. Relative proportion of reads **(A)** and unique peptides (spectral counts normalized by protein length and number of proteins per genome for each sample) **(B)** mapped to a database of metagenomes derived from dominant gut colonizers in a preterm infant. Abbreviations for each organism begin with UC1 (University of Chicago, study code 1), followed by the organism name: CIT, *Citrobacter*; CITii, *Citrobacter* minor strain; CITp, *Citrobacter* plasmid; ENC, *Enterococcus;* ENCp, *Enterococcus* plasmid; ENCv, *Enterococcus* phage; SER, *Serratia*.

To survey the microbial community, we compared results from read and peptide mapping to the metagenomics derived database. Read mapping results confirmed the dominance of *UC1SER*, with lower abundances of *UC1CITs* and *UC1ENC* (Morowitz et al., 2010). Even when the *UC1CITs* are taken together, *Citrobacter* reads are less abundant than *Serratia*. Interestingly, the matched proteomic data indicate that, in combination, the *Citrobacter* account for the largest proportion of the proteome, suggesting that the activity level of these organisms is higher than that of *UC1SER* and *UC1ENC*. The apparent difference in cell abundance compared to activity, based on differences between read mapping and peptide mapping, is most pronounced on DOL 18. For this sample, the read count data indicate *UC1SER* comprised ~60% of the community but its proteins only accounted for 35% of the community proteome (Figure 1).

### Aerobic and anaerobic respiration

**Figure 2:**
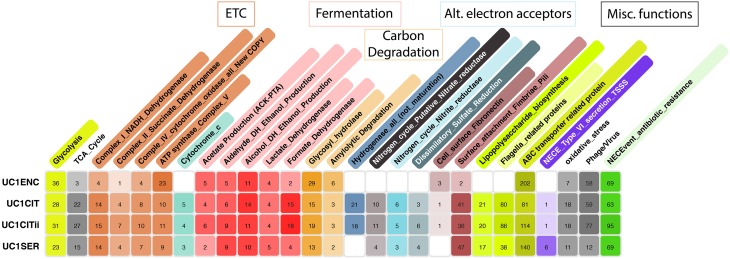
**Metabolic potential of microbes colonizing a preterm infant gut**. ggKbase lists illustrate the broad metabolic potential of microbes colonizing a preterm infant in the first month of life. Numbers in each cell are the number of features per organism per list. Colors per cell are generated in a heat map fashion, with darker colors representing more features per list relative to other organisms.

**Figure 3:**
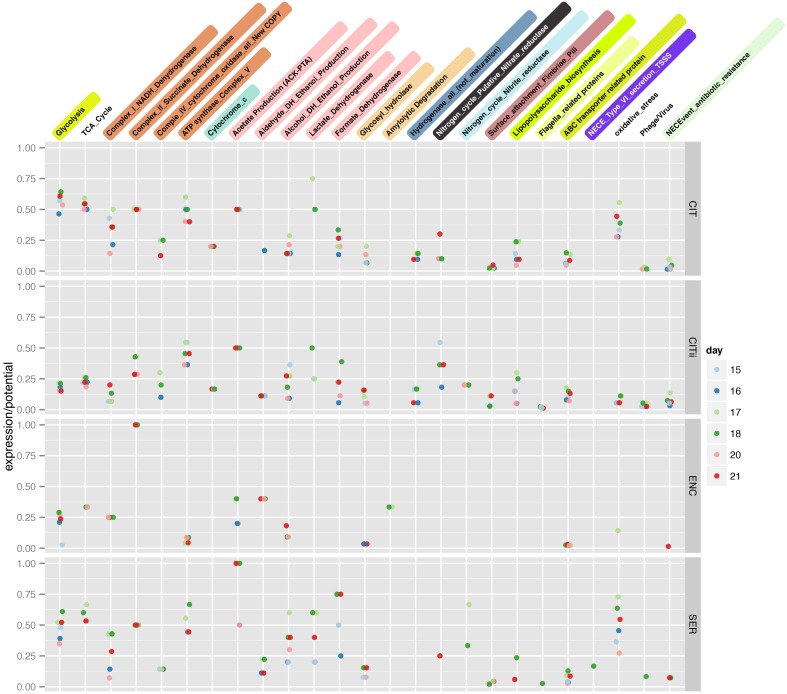
**Expression over potential (genomic content) ratio of infant gut microbes**. A non-redundant count of the number of features identified via proteomics in a metabolic ggKbase list was divided by the number of features in that list and plotted for each organism across time.

During the latter phase of colonization, the infant was supplied with infant formula. Lactose, an abundant constituent in infant formula, can be respired aerobically or fermented. During the period of formula feeding, the community was dominated by *Serratia* and *Citrobacter* strains. These species can grow both aerobically and anaerobically, and capacities for both growth modes are encoded in the genomes of the *Citrobacter* and *Serratia* strains studied here (Figure 2). Evidence for respiration-based metabolism includes detection of proteins from the essentially complete TCA cycle (Supplemental Figure 1). A more direct, and stronger indicator of aerobic respiration, is the identification of most enzymes of the electron transport chain, including multiple terminal oxidases (Figure 3). Previous studies investigating the role of aerobic and anaerobic respiration in the gut of mouse a model found deletion of either ATP synthase or cytochrome bd oxidase, both detected here, was critical to gut colonization (Jones et al., 2007). The aerobic growth pathway was operational throughout this colonization phase in both organisms. Given that the likely source of O_2_ is the intestinal tissue, and that the O_2_ gradient decreases toward the lumen (Albenberg et al., 2014), it seems likely that *UC1CITs* and *UC1SER* growing aerobically are localized toward the mucosa.

Products of glycolysis could also be respired anaerobically, given the presence of pathways for nitrate, nitrite, and sulfate reduction in the genomes (Figure 2). The mass spectrometry measurements identified many *Citrobacter* enzymes likely involved in anaerobic respiration, including proteins from all of these pathways, excluding sulfate reduction (Figure 3). The *Serratia* proteome also included proteins associated with many of these functions. Also expressed are genes involved in the anaerobic reduction of dimethyl sulfoxide and formate. Nitrate reductase proteins were particularly abundant across all time points; this was more pronounced for *UC1CITs* but still detectable in *UC1SER*. The source of nitrate may be the host's immune response through various inflammatory pathways. VLBW preterm infants are both qualitatively and quantitatively immunodeficient, resulting in an exaggerated inflammatory immune response due to their maturing immune system (Gritz and Bhandari, 2015). Nitrate availability in the gut has been shown to give Enterobacteriaceae a fitness advantage over obligate anaerobes (Winter et al., 2013). Citrobacter also expresses nitric oxide dioxygenase, which is involved in aerobic detoxification of NO, presumably protecting the bacterium (and the community) from various toxic nitrogen compounds.

### Fermentation pathways

As with the human milk oligosaccharides they mimic, formula oligosaccharides can be fermented to short chain fatty acids (SCFAs). The genomes of all microorganisms present in the third phase of colonization encode a variety of fermentation pathways and there is clear proteomic evidence for fermentation-based metabolism in both *Citrobacter* strains, the *Serratia* strain, and *Enterococcus faecalis*. These pathways generate SCFAs that are likely absorbed by the infant.

A particularly abundant pathway in *Citrobacter* for which proteins were detected was for the fermentation of fucose to propionate. L-fucose isomerase, the first enzyme needed to degrade L-fucose to L-fuculose, was identified in two samples. The adjacent gene, L-fuculokinase, responsible for conversion of to 1-fucose 1-phosphate, was not identified. L-fucose aldolase, also encoded in this region, converts 1-fucose 1-phosphate to L-lactaldehyde; this protein was also not identified, but the adjacent fructose operon regulator was identified in one sample.

Although both *UC1CITs* have pathways for the anaerobic degradation of rhamnose as well as fucose, the genes for rhamnose degradation (and transport) were not detected.

The protein encoded by the next gene in the anaerobic fucose degradation pathway converts L-lactaldehyde to 1,2-propanediol (1,2-PD). This protein was identified in all samples. *Citrobacter* also can convert 1,2-PD to propionyl-CoA, and probably does so within a well-characterized organelle (a microcompartment), which prevents the accumulation of toxic aldehyde intermediate (Kerfeld and Erbilgin, 2014). Shell proteins for this microcompartment, specifically shell protein PduA, were consistently identified in samples from most days. Propionyl-CoA is likely degraded to propionyl phosphate then to propionate (a SCFA) as propionate kinase, the final enzyme that converts propyionyl phosphate to propionate, was consistently identified in both *UC1CITs*. Propionate may then be excreted, likely to be absorbed by the infant (Tan et al., 2014). Interestingly, *Serratia* does not appear to have the capacity to ferment either fucose or rhamnose, which may be the metabolic basis of their niche separation.

Another prominent fermentative pathway found in the *Citrobacter* proteomes involves enzymes that degrade glycerol, several of which are vitamin B_12_-dependent. Production of vitamin B_12_ (cobalamin) is unique to bacteria and archaea, and is an essential cofactor for many forms of life. We consistently detected proteins required for the biosynthesis of vitamin B_12_, specifically CbiG and CbiK, from *UC1CIT*. The *UC1CITs* are the only relatively abundant organisms in the infant's gut that encode cobalamin biosynthesis genes, and consistent expression of this pathway suggests it to be a key role in the community (Supplemental Figure 1). Notably, these cobalamin biosynthesis enzymes operate under anaerobic conditions, a further indication of anaerobic niches in the gut during this phase of colonization.

Additionally, enzymes were identified for a fermentation pathway that converts glycerol to 1,3-propanediol (1,3-PD) and other SCFA by-products (using the glycerol dehydratase complex: EC:4.2.1.30; three subunits, all of which were identified by proteomics in all samples). *Citrobacter* is one of a small number of bacterial genera with the glycerol fermentative pathway (others include *Klebsiella*, *Clostridium*, and *Lactobacillus*). The SCFA by-products of this pathway are acetate and sometimes butyrate (Abbad-Andaloussi et al., 1996). For the latter, the enzymes required for acetyl-CoA conversion to butyrate are poorly maintained within the genomes of each of these genera, and only select strains contain them (Louis et al., 2004). *Citrobacter* has the genes to convert acetyl CoA to crotonoyl-CoA (e.g., for amino acid biosynthesis), but lacks those required to form butanoate.

For glycerol breakdown, *Serratia* lacks the glycerol dehydratase complex found in *Citrobacter* (EC:4.2.1.30). However, it has glycerol kinase (EC:1.1.1.6) and glycerone kinase (EC: 2.7.1.29), allowing it to convert glycerol to glycerone phosphate, potentially for consumption via glycolysis. Both of these enzymes were identified by proteomics, although only in the day 21 sample. *Serratia* also has glycerophosphoryl diester phosphodiesterase (EC:3.1.4.46) that converts alpha glycerophosphodiester to *sn*-glycerol-3-phosphate. This is also the product of glycerol kinase (EC:2.7.1.30, which was identified by proteomics in all samples). The *sn*-glycerol-3-phosphate can be degraded by glycerol-3-phosphate dehydrogenase (EC: 1.1.5.3) to dihydroxyacetone phosphate (glycerone phosphate), and some of these proteins were identified. *Serratia* then combines *sn*-glycerol-3-phosphate with acyl CoA to form 1-acyl-sn-glycerol 3-phosphate (identified by proteomics in one sample). These, and other proteins, are likely redirected for use in lipid biosynthesis.

We also identified multiple *Serratia* proteins of the inositol degradation pathway, including inositol 2-dehydrogenase, myo-inositol catabolism protein IolH, inosose dehydratase, 3D-(3,5/4)-trihydroxycyclohexane-1,2-dione hydrolase, and 5-dehydro-2-deoxygluconokinase. The strong representation of these enzymes indicates a potentially important role for *Serratia* in degradation of this compound, which is an important component of both breast milk and infant formula (Hallman et al., 1992). *Citrobacter* also has some enzymes for inositol degradation. However, the identification of only two proteins from the *Citrobacter* pathway may indicate that inositol is a less important substrate for this organism compared to *Serratia*.

### Motility, toxicity, and invasion

Several pathways enable microorganisms to cope with the gut immune system, respond to administered antibiotics, and to manage compounds produced by other microorganisms. Catalase, an enzyme used to protect cells from reactive oxygen species (ROS) by degrading hydrogen peroxide to water and oxygen, is consistently found in both *UC1CITs* and *UC1SER* on most days. Hydrogen peroxide can be produced by the intestinal epithelium and neutrophils during inflammation response, along with other ROS (Winter et al., 2013). Other protective antioxidant proteins such as lipid hydroperoxide peroxidase, alkyl hydroperoxide reductase, superoxide dismutase, and glutathione peroxidase were identified in samples collected on most days, and were particularly abundant in *UC1CITs* and *UC1SER*.

Often ROS exposure occurs in close proximity to the host epithelium (Winter et al., 2013). The ability to move away from ROS and toward a more favorable environment seems critical for the microbes studied here. We identified many *UC1CITii* polar flagella-related proteins in samples collected on several days; no lateral flagella proteins were identified and flagella proteins were not identified for *UC1CIT* (Figure 3). Proteins for twitching motility were detected for *UC1CITii*. Additionally, proteins for chemotaxis, specifically chemotaxis protein methyltransferase CheR, are detected on all days in *UC1CITii*. Chemotaxis related proteins were not detected in the dominant strain, or *UC1SER*, possibly suggesting a more planktonic state for the minor strain. Perhaps the increased motility allows the minor strain to escape ROS, as expression levels and frequency of oxidative stress related genes are lower in the minor strain (Figure 3).

In contrast to chemotaxis and flagellar movement, proteins for biofilm formation and many fimbrial attachment proteins are detected in *UC1CIT* and *UC1SER*, though these are also detectable in *UC1CITii*. Type-1 fimbrial proteins, associated with capacity to attach to host gut epithelium (Juge, 2012), were detected consistently across the time series, as were proteins for biofilm regulation and formation. Transcriptional regulator and periplasmic proteins csgD and csgF, involved in curli biosynthesis, were also detected on 1 day in *UC1CITii* (Barnhart and Chapman, 2006). It is unclear whether curli expression in *UC1CITii* is promoting adherence to one another or host adherence, as it is capable of both (Barnhart and Chapman, 2006). *Citrobacter's* affinity for host fucosylated glycans would suggest colonization of the mucosa.

Proteins that respond to both host and inter-species bacterial attack, such as the type VI secretion system (T6SS) in *UC1SER*, were detected on several days. T6SS were first investigated for their role in pathogenesis (Jani and Cotter, 2010), but have been studied more recently for “T6SS-dueling,” a mechanism in which the T6SS can kill and outcompete neighboring microbes for resources (Basler et al., 2013). Proteins involved in production of bacteriocin toxins like colicin were also detected on most days, but only from *U1CITii*. Many gram-negative bacteria can produce a variety of bacteriocins that can target closely related species or strains that occupy similar niches (Kleanthous, 2010).

A competitive advantage for niche space (and persistence in the hospital environment) is also gained through various antibiotics resistance mechanisms. A variety of antibiotic resistance proteins were detected for all organisms across the time series, but most frequently in the *UC1CITs*. Most consistently detected for all organisms were efflux pumps. Efflux pumps can remove various toxins, waste products, and antibiotics (Fernández and Hancock, 2012). It was speculated that the multipurpose functionality of such pumps could aid in survival, both in and out of the host, as antibiotics and various biocides are encountered in both environments (Brooks et al., 2014).

### Comparison of major and minor citrobacter strains

The two *UC1CITs* strains share an approximate 98.96 and 99.23% nucleotide and amino acid identity between their orthologs, respectively. Read mapping data confirms genome abundances previously published (Morowitz et al., 2010), with a drop in the minor strain population around DOL 18 (Figure 1). Interestingly, metabolic activity, as reflected in the proteome composition, does not correlate closely with genome abundance, as no decrease in protein spectral counts mapping to the minor strain occurs (Figure 1). This could reflect steady state growth of the abundant *Citrobacter* strain and rapid growth of the minor strain. This result highlights the value of proteomic data for uncovering aspects of the dynamics not apparent from genome abundance information alone. Difference between genome abundance (from 16S rRNA gene surveys or metagenomics) and microbial activity levels have been reported previously. Different methodologies can distinguish living from dead or inactive cells. For example, Maurice et al. (2013) distinguished subgroups based on nucleic acid concentrations and lack of membrane integrity. However, Franzosa et al. (2014) showed that RNA and DNA abundances were generally well-correlated, with the exception of a few select pathways. Erickson et al. (2012) reported varying degrees of incongruence between organisms based on mapped metagenomic reads vs. peptide spectral counts in patients with Crohn's disease. To our knowledge, no genome abundance to protein expression difference, as detected here, has been reported for gut-associated microorganisms in infants.

**Figure 4:**
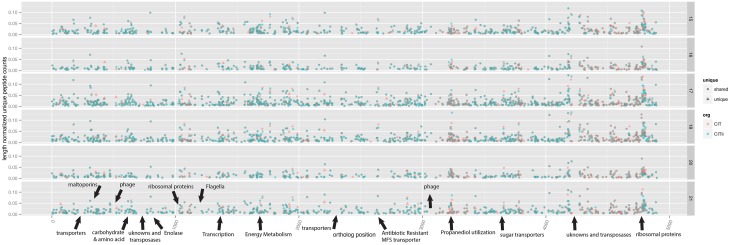
**Comparison of proteomic profiles of two closely related** ***Citrobacter*** **strains**. Unique peptide counts, normalized by the length of the protein, were mapped to *UC1CITs'* contigs (aligned on the x-axis). Panels are separated by day of life. For a description of ggKbase lists, see the Materials and Methods and Supplemental File 1. Triangles represent genes unique to the respective strain. Annotations marked with arrows indicate features of interest.

We looked for differences in overall proteome composition for the *Citrobacter* strains, and for evidence for the production of proteins unique to one of the strains. Spectral coverage across the genomes was relatively complete, and generally, expression of the minor strain tracked with the major stain (Figure 4). Notable exceptions were the flagella and chemotaxis-related proteins, as noted above, and drops in coverage often associated with phage related regions, mobile elements, and some regions associated with transport and membrane proteins. Interestingly, several unique genes, genes encoded in one strain and not the other, were expressed. There are 233 and 84 genes not shared with the other strain in *UC1CIT* and *UC1CITii*, respectively, and 25 and 10 of these proteins were detected via proteomics for *UC1CIT* and *UC1CITii*, respectively. These span broad functions from transcription, translation, and metabolism related proteins. From the major strain, novel proteins were identified on most days, such as carbamoyl phosphate synthase involved in pyrimidine and amino acid metabolisms, an alcohol dehydrogenase used in ethanol production, a transporter for glutathione binding, and an aromatic amino acid aminotransferase. Carbamoyl phosphate synthase is the rate-limiting step in L-arginine production and has been linked to an increase of NEC in preterm infants (Watkins and Besner, 2013). In the minor strain, ABC transporters and a DNA translocase FtsK variant involved in cell division and chromosome separation were detected on most days. In combination, the differences in proteome composition support the inference that the *Citrobacter* strains occupy distinct niches.

## Concluding remarks

The VLBW infant gut microbiome is relatively uncharacterized and little is known about microbial metabolism during the critical first few weeks of life. The opportunity for organisms to grow via both aerobic and anaerobic respiration might be anticipated to develop over the time period in which GI tract transitions from an aerobic to anaerobic state. However, the range of aerobic and anaerobic metabolisms detected at the same time may suggest heterogeneity in the developing gut in which facultative anaerobes are likely to dominate. Different niches may be associated with sub-populations of *Serratia* and the two *Citrobacter* strains in different gut environments. Further, metabolic differences between the *Citrobacter* strains support the suggestion that the populations occupy distinct niches. The distinct differences in inferred abundances and activity levels for these strains likely reflect changing opportunities occurring during this colonization phase.

## Author contributions

JB, MM, and RH conceived of the project. MM organized cohort recruitment and organized sample collections. BB conducted the strain-level genome curation and prepared the metagenomic database. JY, and RH facilitated and ran the proteomics samples. BB, RM, and JB analyzed the data and wrote the final manuscript. All authors have read and approve the manuscript.

### Conflict of interest statement

The authors declare that the research was conducted in the absence of any commercial or financial relationships that could be construed as a potential conflict of interest.

## Supplementary material

Supplemental Figure 1 **Expression over potential (genomic content) ratio of infant gut microbes**. A non-redundant count of the number of features identified via proteomics in a metabolic ggKbase list was divided by the number of features in that list and plotted for each organism across time. Figure 3 in the main text is a subset of ggKbase lists from this figure.

## Abbreviations

GIT: gastrointestinal tract
VLBW: very low birth weight
NICU: neonatal intensive care unit
DOL: days of life
c-section: cesarean section
NEC: necrotizing entercolitis
nano-2D-LC-MS/MS: nanospray-two dimensional liquid chromatography coupled with tandem mass spectrometry
SCFA: short-chain fatty acid
1,2-PD: 1,2-propanediol.
