# Supplementary figures and images for: Strain-resolved microbial community proteomics reveals simultaneous aerobic and anaerobic function during gastrointestinal tract colonization of a preterm infant
Source: Front Microbiol. 2015 Jul 1;6:654. doi: 10.3389/fmicb.2015.00654 (PMC4487087; doi:10.3389/fmicb.2015.00654)

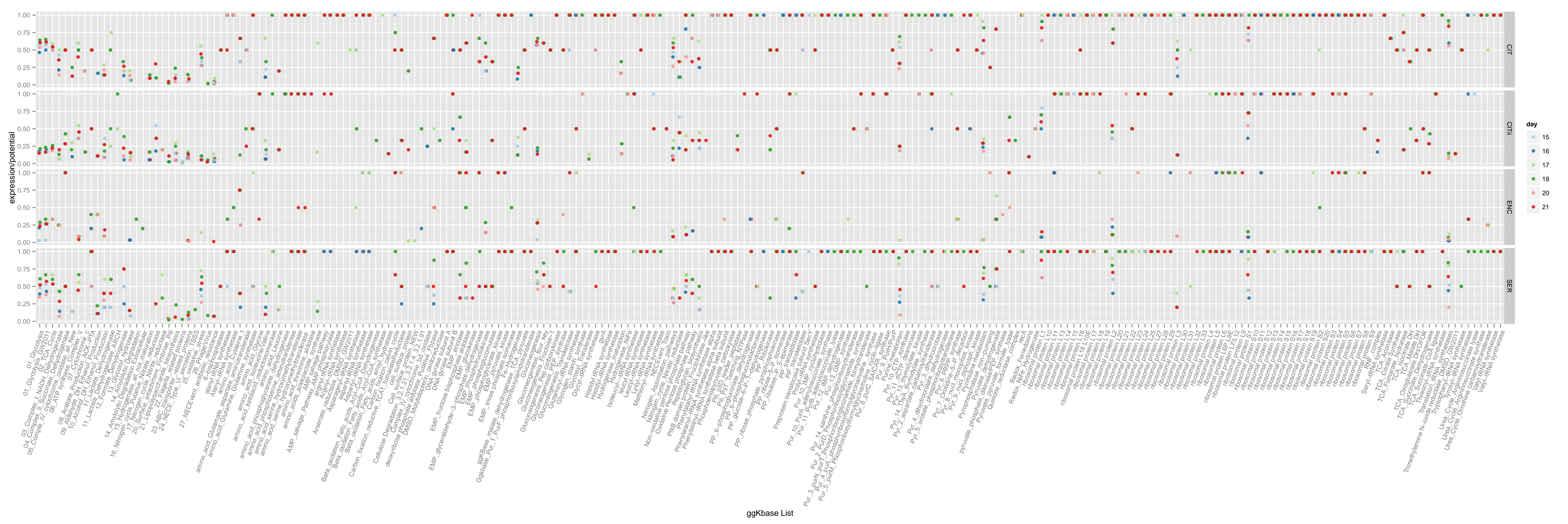

Supplement: Supplemental Figure 1 — Expression over potential (genomic content) ratio of infant gut microbes. A non-redundant count of the number of features identified via proteomics in a metabolic ggKbase list was divided by the number of features in that list and plotted for each organism across time. Figure 3 in the main text is a subset of ggKbase lists from this figure. [file Image1.PDF]
